# Mortality Risk for Acute Cholangitis (MAC): a risk prediction model for in-hospital mortality in patients with acute cholangitis

**DOI:** 10.1186/s12876-016-0428-1

**Published:** 2016-02-09

**Authors:** Jochen Schneider, Alexander Hapfelmeier, Sieglinde Thöres, Andreas Obermeier, Christoph Schulz, Dominik Pförringer, Simon Nennstiel, Christoph Spinner, Roland M. Schmid, Hana Algül, Wolfgang Huber, Andreas Weber

**Affiliations:** II. Medizinische Klinik und Poliklinik, Klinikum rechts der Isar, Technische Universität München, München, Germany; Institute of Medical Statistics and Epidemiology, Technische Universität München, München, Germany; Klinik für Orthopädie, Labor für Infektionsforschung, Klinikum rechts der Isar, Technische Universität München, München, Germany; Institut für klinische Chemie und Pathobiochemie, Klinikum rechts der Isar, Technische Universität München, München, Germany; I. Chirurgische Klinik und Poliklinik, Klinikum rechts der Isar, Technische Universität München, München, Germany

**Keywords:** Bacterial infections, Cholangitis, Gastroenterology

## Abstract

**Background:**

Acute cholangitis is a life-threatening bacterial infection of the biliary tract. Main focus of this study was to create a useful risk prediction model that helps physicians to assign patients with acute cholangitis into different management groups.

**Methods:**

981 cholangitis episodes from 810 patients were analysed retrospectively at a German tertiary center.

**Results:**

Out of eleven investigated statistical models fit to 22 predictors, the Random Forest model achieved the best (cross-)validated performance to predict mortality. The receiver operating characteristics (ROC) curve revealed a mean area under the curve (AUC) of 91.5 %. Dependent on the calculated mortality risk, we propose to stratify patients with acute cholangitis into a high and low risk group. The mean sensitivity, specificity, positive and negative predictive value of the corresponding optimal cutpoint were 82.9 %, 85.1 %, 19.0 % and 99.3 %, respectively. All of these results emerge from nested (cross-)validation and are supposed to reflect the model’s performance expected for external data. An implementation of our risk prediction model including the specific treatment recommendations adopted from the Tokyo guidelines is available on http://www2.imse.med.tum.de:3838/.

**Conclusion:**

Our risk prediction model for mortality appears promising to stratify patients with acute cholangitis into different management groups. Additional validation of its performance should be provided by further prospective trails.

## Background

Acute cholangitis is a life-threatening bacterial infection of the biliary tract ranging from mild symptoms such as fever and/or chills, abdominal pain and jaundice to septic shock [1–5]. In general, an obstruction of the biliary system results in a disruption or a reduction of the bile flow into the duodenum and leads to a biliary stasis. Bacteria reach the biliary system either by ascent from the intestine or by the portal venous system [1]. Inside this biliary stasis the bacteria can multiply to a great extent [1]. Furthermore, the biliary stasis leads to an increased intrabiliary pressure with cholangiovenous reflux and bacteraemia [1]. The most common cause of cholangitis is choledocholithiasis [5, 6]. The key elements of therapy in acute cholangitis are adequate antimicrobial treatment to avoid or manage septic complication and biliary decompression to restore biliary drainage in case of obstruction [3, 6]. In severe cholangitis, an early interventional approach is absolutely essential for survival [3]. However, cholangitis manifests from a broad variety of causes and with varied severity and therefore many factors might have an influence on the survival in patients with acute cholangitis [4]. Major aim of the study was to create a feasible risk model for predicting mortality in patients with acute cholangitis, giving the physicians an orientation how fast biliary decompression has to be performed and how potent antimicrobial treatment should be. In the present study, the mortality rate of patients with acute cholangitis was analysed and related to clinical, laboratory and etiological factors.

## Methods

### Study population

All patients were admitted to the II. Medizinische Klinik und Poliklinik, Klinikum rechts der Isar, Technische Universität München. All patients included to the study had definitive cholangitis according to the Tokyo guidelines 2013 (TG13). Patients were recruited as follows: First, our clinical endoscopic database was queried for patients who underwent an endoscopic retrograde cholangiography (ERC) or a percutaneous transhepatic cholangiography (PTC). Then, clinical indications for the endoscopic or percutaneous procedures were clarified. For inclusion to the study, all patients underwent endoscopic retrograde cholangiography (ERC) or percutaneous transhepatic cholangiography (PTC) due to biliary obstruction. Patients had to present elevated temperature (temp > 38 °C) and/or elevated infection parameters (leukocytes > 12 G/L and/or C-reactive protein > 3 mg/dL) as well as elevated cholestasis parameters (1.5 times of the upper limit of the normal range in bilirubin and/or 2.5 times of the upper limit of the normal range in γ-glutamyltransferase and/or 2.5 times of the upper limit of the normal range in alkaline phosphatase). Data collection and analysis of patients with incomplete data records (missing laboratory values or medical examination), end stage liver cirrhosis as well as other focus of infection was not performed. 13 patients died due to other causes than cholangiosepsis and were excluded from the study. These patients died either from end-stage malignancy or complications such as bleeding.

### Assessment of predictors for mortality in univariate analysis

Definition of organ failure was mainly based on the Tokyo guidelines [3]. One of the following criteria needed to be fulfilled: hypotension requiring catecholamine, serum creatinine > 2 mg/dl, quick’s value < 50 %, platelet count < 100.000 /mm^3^ and disturbance of consciousness. Age was dichotomised in two ways, patients ≥ 65 years of age and patient’s ≥ 75 years of age. The chronological age of ≥ 65 years is a well-accepted definition of elderly or older person in medicine [7–10]. The dichotomisation of age (≥75 years) and other predictors like fever (temperature ≥ 39 °C), hyerbilirubinemia (≥5 mg/dL) were derived from the Tokyo guidelines [3].

### Statistical analysis

Data analysis was performed with the statistical analysis tool R 3.1.0 (R Foundation for Statistical Computing, Vienna, Austria).

#### Univariate analysis

Fisher’s Exact test was used to investigate the association of binary and dichotomized predictor variables to in-hospital mortality. Corresponding Odds Ratios and 95 % confidence intervals are presented. All statistical tests were performed on an exploratory, two-sided 5 % significance level.

#### Multivariate analysis

Eleven statistical models were investigated to find the one that predicts mortality best. The models were 1) logistic regression with stepwise variable selection based on Akaike’s Information Criterion (AIC) [11], 2) logistic regression with stepwise variable selection based on the Bayesian Information Criterion (BIC) [11], 3) cross-validated generalized linear models with L2 (ridge) penalties [12], 4) cross-validated generalized linear models with L1 (lasso) penalties [12], 5) gradient boosting with component-wise linear models [13], 6) conditional inference classification trees [14], 7) conditional inference Random Forest [15], 8) conditional inference Random Forest with test-based variable selection [16, 17], 9) conditional inference Random Forest with test-based variable selection and Bonferroni adjustment for multiple testing [16, 17], 10) gradient boosting with regression trees [13], 11) support vector machines (SVM) with parameters tuned by cross-validation [18].

These model were fit to the following 22 predictors:

- Age as continuous variable
- Age as dichotomised (age ≥ 65) variable
- Age as dichotomised (age ≥ 75) variable
- Temperature as continuous variable
- Temperature as dichotomised (temperature ≥ 39 °C) variable
- Leucocytes as continuous variable
- Leucocytes as dichotomised variable (leucocytes > 15 G/l)
- Bilirubin as continuous variable
- Bilirubin dichotomised (bilirubin ≥ _5 mg/dl) variable
- Quick’ s value as continuous variable
- Quick’ s value as dichotomised (quick’s value < 50 %) variable
- Serum creatinine as continuous variable
- Serum creatinine as dichotomised (serum creatinine ≥ 2 mg/dl) variable
- Platelet count as continuous variable
- Platelet count as dichotomised (platelet count < 100 G/L) variable
- Sex
- Mental confusion
- Previous cholangitis episode
- Previous intervention (ERC/PTC)
- Hypotension requiring catecholamine
- Underlying cause of cholangitis (malignant/non-malignant)

As we seek to identify the model that will perform best on external data and as we would like to learn about its expected predictive performance, there needs to be a validation. In a data-rich situation the data is simply split into three parts, a training set, a validation set and a test set [19]. The models are fit to the training set and applied to the validation set to identify the best model. This best model is then applied to the test set to assess the performance it can achieve on unseen data. As we are not in a data-rich situation, nested five-fold cross-validation with an internal and external loop was applied alternatively. Here the data is split into five parts. Each part serves as a test set for a model found to perform best on the remaining four parts of the data (= external cross-validation loop). The latter parts are again split into five parts, each one makes up a validation set while the remaining four are used as training set (= internal cross-validation loops). The performance of the best models found in the internal cross-validation loops can then be computed on the test data of the external cross-validation loop. This is the performance that can be expected for the application of a best model to unseen data (= model assessment). The best model itself is determined on the external cross-validation loop (= model selection). It is finally fit to the entire data to use all of the information available for learning.

The optimal cutpoints used to categorize patients into a high and low risk group were defined to be the ones maximising the Youden-Index = Sensitivity + Specificity – 1. They were computed within the external cross-validation loop, as the test sets of the data were omitted. Estimates for sensitivity, specificity, positive predictive value and negative predictive value were finally assessed on the test sets.

## Results

### Patients’ characteristics

981 acute cholangitis episodes from 810 patients were included to the study. Median age was 68 years (range 24 – 97 years). Underlying causes for acute cholangitis are illustrated in Table 1. In most cases, acute cholangitis was due to malignant diseases. All patients underwent either endoscopic retrograde cholangiography or percutaneous transhepatic cholangiography. PTC was performed in 310 cholangitis episodes. Overall, mortality rate was 4.5 %. These patients died due to cholangiosepsis.

**Table 1:** Patients’ baseline characteristics

Baseline characteristics	
number of patients	810
- median age in years (range)	68(27-97)
- male	456
number of cholangitis episodes	981
underlying cause of cholangitis episodes	
malignant genesis:	509 (51,9 %)
- cholangiocarcinoma	206 (21,0 %)
- pancreas cancer	162 (16,5 %)
- hepatocellular carcinoma	7 (0,7 %)
- liver metastases with intrahepatic biliary obstruction:	
o colorectal cancer	50 (5,1 %)
o gastric cancer	30 (3,1 %)
o gallbladder cancer	28 (2,9 %)
o other malignant tumors (breast cancer, oesophagus cancer…)	26 (2,7 %)
benign genesis:	391 (39,9 %)
- stricture of the biliodigestive anastomosis	32 (3,3 %)
- chronic pancreatitis with extrahepatic biliary obstruction	17 (1,7 %)
- bile duct stricture after cholecystectomy	36 (3,7 %)
- liver cyst with intrahepatic biliary stricture	1 (0,1 %)
- adenoma of the papilla vateri	6 (0,6 %)
- choledocholithiasis	271 (27,6 %)
- primary sclerosing cholangitis	21 (2,1 %)
- trauma associated strictures	6 (0,6 %)
- secondary sclerosing cholangitis	1 (0,1 %)
idiopathic biliary stricture:	81 (8,3 %)

### Univariate analysis of predictors for mortality

Univariate analysis of different predictors is illustrated in Table 2. Organ failure showed by far the strongest association with mortality [P < 0.001, Odds Ratio (OR): 47.1, 95 % confidence interval (95 % CI): (14.3 -155.1)]. Among predictors contributing to organ failure, the highest OR was observed for mental confusion [P < 0.001, OR: 37.6, 95 %-CI: (16.7 - 84.9)], followed by hypotension requiring catecholamine [P < 0.001, OR: 7.7, 95 %-CI: (3.6- 16.5)], quick’s value < 50 % [P < 0.001, OR: 6.4. 95 %-CI: (2.9 - 14.5)], serum creatinine >2 mg/dl [P = 0.012, OR: 4.3, 95 %-CI: (1.5 - 11.8)] and a platelet count < 100.000 /mm^3^ [P < 0.038, OR: 3.0, 95 %-CI: (1.1- 8.2)] before a treatment was initiated. In addition, a bilirubin level ≥ 5 mg/dl [P < 0.001, OR: 4.5, 95 %-CI: (2.2 - 9.9)], leucocytes ≥ 15 g/l [P = 0.026, OR: 2.3, 95 % CI: (1.1 - 4.7)], the presence of bacteraemia[P = 0.004, OR: 2.8, 95 %-CI: (1.4 - 5.6)], insufficient drainage [P < 0.001, OR 4.5, 95 %-CI: (2.2-9.1)], aspartat-Aminotransferase (AST) > 70 [P = 0.046,OR 2.1, 95 %-CI: (1.0-4.3)] and PTC as therapeutic approach [P = 0.011, OR: 2.4, 95 %-CI: (2.2 - 9.0)] were also significantly associated with a higher mortality rate. Concerning etiological factors, patients with a malignant underlying disease had also a higher incidence of mortality compared to patients with an idiopathic or benign underlying disease for cholangitis [P = 0.001, OR: 3.0, 95 %-CI: (1.4 - 6.4)]. A direct comparison of risk predictors by the OR is possible in this case as they are measured on the same scale, i.e., they are binary or dichotomized.

**Table 2:** Univariate analysis of binary and dichotomized risk predictors

Univariate analysis of binary and dichotomized risk predictors	Incidence of mortality	[Odds ratio (95 %-CI)] *P*- value
age ≥ 65	4.0 %(23/579)	[1.1(0.5-2.3)] P = 0.736
age < 65	3.5 %(14/402)	
age ≥ 75	4.3 %(13/303)	[1.2(0.6-2.5)] P = 0.588
age < 75	3.5 %(24/678)	
male	2.9 %(16/549)	[1.7(0.8-3.4)] P = 0.129
female	4.9 %(21/432)	
fever (temp ≥39 °C)	3.8 %(2/52)	[1.0(0.2-4.4)] P = 1.000
fever (temp <39 °C)	3.8 %(34/905)	
previous episode of cholangitis	3.8 %(17/446)	[1.0(0.5-1.9)] P = 1.000
no previous cholangitis episode	3.7 %(20/535)	
indwelling stent (stent therapy)	3.4 %(16/466)	[0.8(0.4-1.7)] P = 0.619
no indwelling stent (stent therapy)	4.1 %(21/515)	
PTC as interventional approach	6.1 %(19/310)	[2.4(1.2-4.6)] P = 0.011
no PTC as interventional approach	2.7 %(18/671)	
mental confusion^a^	25.9 %(29/112)	[37.6(16.6-85.0)] P < 0.001
no mental confusion	0.9 %(8/869)	
previous intervention (ERC/PTC)	5.0 %(19/383)	[0.6(0.3-1.2)] P = 0.125
no previous intervention	3.0 %(18/598)	
hypotension requiring catecholamine^a^	18.3 %(11/60)	[7.7(3.6-16.6)] P < 0.001
no hypotension requiring catecholamine	2.8 %(26/921)	
insufficient drainage	8.3 %(20/240)	[4.5(2.2-9.1)] P < 0.001
adequate drainage	2.0 %(14/706)	
organ failure including all parameters with^a^	15.7 %(34/217)	[47.1(14.3-155.2)] P < 0.001
no organ failure	0.4 %(3/764)	
leucocytes > 15 G/l	6.9 %(12/174)	[2.3(1.1-4.8)] P = 0.026
leucocytes ≤ 15 G/L	3.1 %(25/807)	
bilirubin ≥5 mg/l	6.4 %(29/452)	[4.5(2.0-9.9)] P < 0.001
bilirubin <5 mg/l	1.5 %(8/529)	
aspartat-Aminotransferase (AST) > 70	5.3 %(19/357)	[2.1(1.0-4.3)] P = 0.046
aspartat-Aminotransferase (AST) ≤ 70	2.6 %(14/532)	
alanin-Aminotransferase (ALT) > 70	3.0 %(14/462)	[0.7(0.3-1.3)] P = 0.242
alanin-Aminotransferase (ALT) ≤ 70	4.6 %(23/502)	
quick’s value < 50 %	16,7 %(9/54)	[6.4(2.8-14.5)] P < 0.001
quick’s value ≥ 50 %	3.0 %(28/927)	
serum creatinine >2 mg/dl^a^	13.2 %(5/38)	[4.3(1.5-11.8)] P = 0.012
serum creatinine ≤ 2 mg/dl	3.4 %(32/943)	
platelet count < 100.000/mm3^a^	9.8 %(5/51)	[3.0(1.1-8.2)] P = 0.038
platelet count ≥ 100.000/mm3	3.4 %(32/930)	
bacteremia	7.7 %(14/182)	[2.8(1.4-5.6)] P = 0.004
no bacteremia	2.9 %(23/799)	
malignant genesis	1.9 %(9/472)	[3.0(1.3-6.5)] P = 0.004
non malignant genesis	5.5 %(28/509)	

^a^parameters included to organic failure

### Multivariate risk prediction model for in-hospital mortality

Only predictors that are ,in general, easily to assess at the time point of hospital admission were used for the multivariate risk prediction model including the predictors “creatinine”, “mental confusion”, “bilirubin”, “leucocytes”, “temperature”, “platelet count”, “sex”, “previous cholangitis episodes”, “previous intervention (ERC/PTC)”, “age“, “hypotension requiring catecholamine”, “quick’s value” and “underlying disease”. These 13 predictors have been dichotomized using several established cutpoints and combined to produce further information. In summary, 22 risk predictors were created, which are listed in the *statistical analysis* section.

Eleven statistical prediction models were evaluated and (cross-)validated revealing the best prediction performance for the Random Forest model. This choice leads to some conclusions: There might be non-linear effects and complex relations among predictors which cannot be coped, e.g., with the application of regression models in their basic form. The logistic regression model, for example, led to a decisively worse prediction performance. As all of the models that use variable selection were also inferior, it can be concluded that each predictor has a certain predictive value and is able to beneficially contribute to the prediction model.

**Fig. 1:**
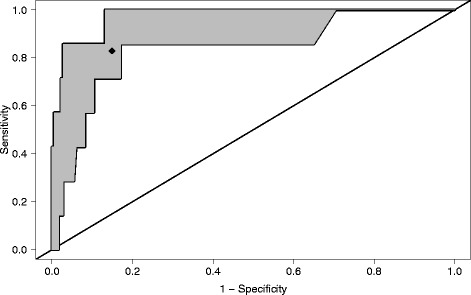
Range of the receiver operating characteristic (ROC) curves obtained by five-fold cross-validation. The mean AUC is 91.5 %. The rhomb indicates the mean performances of the optimal cutpoint

The predictive performance of the Random Forest prediction model was measured by ROC analysis which resulted in a mean AUC of 91.5 %. Fig. 1 illustrates the range of ROC curves of the five (cross-)validation steps. The corresponding AUC values were 84.4 %, 89.3 %, 93.1 %, 93.5 % and 96.9 %. The optimal cut-off point, defined by the maximal Youden-Index, is the predicted mortality risk of 0.7 %. The corresponding mean sensitivity and specificity were 82.9 % (computed from the five cross-validated values 71.4 %, 71.4 %, 85.7 %, 85.7 % and 100 %) and 85.1 % (computed from the values 78.0 %, 82.5 %, 84.1 %, 87.4 % and 93.4 %). Furthermore, the mean positive predictive and negative predictive values of the cut-point were 19 % (computed from 14.9 %, 15.8 %, 17.1 %, 17.9 %, 29.4 %) and 99.3 % (computed from 99.3 %, 99.4 %, 98.8 %, 98.8 %, 100 %). Instead of the mean values one could also chose to use the worst performance values for a conservative assessment of the model.

### Proposed treatment algorithm for prospective evaluation

**Figure 2:**
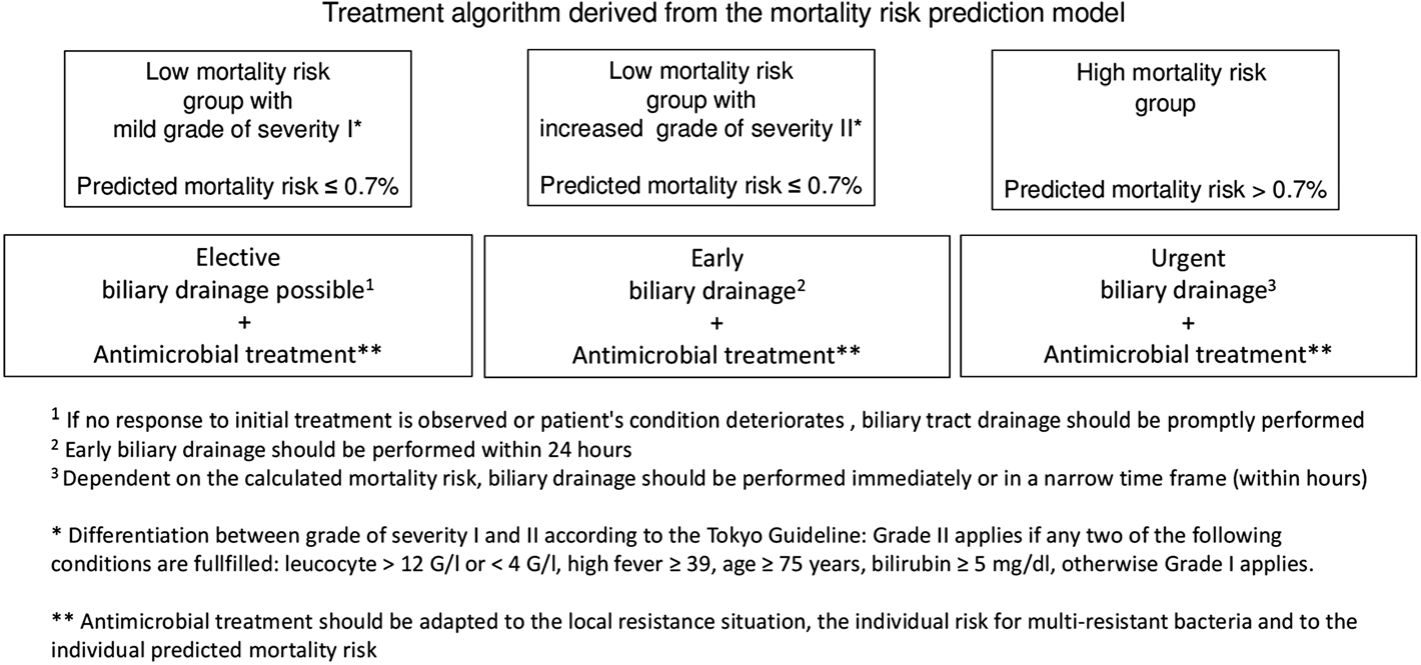
Recommended treatment algorithm based on the calculated mortality risk

Based on the optimal cut-off value (0.7 %) of the prediction model, patients were classified into a high risk and a low risk group (Fig. 2). Referring to the Tokyo guidelines [20], the low risk group was further subdivided into acute cholangitis with a mild (grade I) and a moderate (grade II) grade of severity. According to the Tokyo guidelines, moderate grade of severity applies if any two of the following conditions are fulfilled: Leucocyte > 12 G/l or < 4 G/l, high fever ≥ 39 °C, age ≥ 75 years, bilirubin ≥ 5 mg/dl. Thus, acute cholangitis of the low risk group, which do not fulfil the above mentioned criteria for moderate grade (II) of severity are defined as acute cholangitis with a mild (I) grade of severity. Dependent on the risk groups, the following treatment recommendation are proposed:

#### Low risk mortality group

Elective biliary drainage is recommended for acute cholangitis with a mild grade of severity (according to the Tokyo guidelines). In case of acute cholangitis with a moderate grade of severity (according to the Tokyo guidelines), early biliary (within 24 h) drainage should be performed

#### High risk mortality group

Urgent biliary drainage is recommended for patients with a high predicted mortality risk. Dependent on the level of mortality risk, biliary drainage should be performed either immediately or within a narrow time frame (within hours).

In all three groups, immediate initiation of antimicrobial treatment is recommended. The use of broad spectrum antibiotics should be adapted to the local resistance situation, the individual risk for multi-resistant bacteria and to the individual predicted mortality risk.

### Implementation

Due to its elaborateness, it is not possible to put the prediction model to paper. Therefore, the risk prediction model is available online on http://www2.imse.med.tum.de:3838/. After the user has made his choices for the risk predictors, an individual risk prediction, the membership to a certain risk group and a treatment recommendation is returned for the patient.

## Discussion

Adequate management of acute cholangitis is essential for survival [21–24]. However, acute cholangitis manifests from a broad variety of causes and with varied severity. Therefore, risk stratification in patients with acute cholangitis for increased mortality seems to be reasonable, allowing a differentiated antimicrobial and interventional treatment. In literature, the Tokyo guidelines provide international standards for diagnostic and severity assessment criteria [3] and consider organ dysfunction as the most common predictor of poor outcome. According to the Tokyo guidelines [3], acute cholangitis should be classified into three grades of severity (Grade I – III). A disadvantage of TG13 is that

Patients are equally weighted within the respective severity groups, regardless of the number and type of fulfilled predictors. For example, TG13 does not differentiate between the type and number of organ failures. However, our data strongly suggest that the type of organ failure should be weighted differently for predicting mortality. In the univariate analysis of binary and dichotomized risk predictors, mental confusion was the strongest predictor, compared to renal, liver or haematological disorders. Disturbance of consciousness was also reported to be a well predictor for an increased mortality in other infections like community acquired pneumonia [25]. Similar to our data, mental confusion was associated with the highest risk for mortality in an international validation study [25], assessing the severity of pneumonia: The odds for mortality in patient with mental confusion was 8 times higher compared to patients without mental confusion [OR: 8.1, 95 %-CI (4.8 - 13.7)]. In contrast, other significant risk predictors like respiratory rate > 30/min, low systolic blood pressure < 90 mmhg , urea > 7 mmol/l, albumin < 30 g/dl had odds ratios (95 %-CI) of 1.7(1.1 - 2.8), 2.4(1.4 - 3.8), 5.6(3.1 - 10), 4.9(2.8 - 8.4), respectively. Furthermore, TG 13 uses only dichotomized laboratory parameters. Therefore, TG13’s severity assessment is similar irrespective of whether laboratory parameters differ strongly or only marginally from the determined cut off value. Our prediction model considers patients with strongly or only marginally deviating laboratory parameters differently. To the best of our knowledge, this is the first prediction model for adapting the treatment of acute obstructive cholangitis to the individual calculated mortality risk. In contrast to TG13, the individual mortality risk is assessed by using 22 continuous and dichotomized predictors. Patients are classified into a high risk and a low risk group, based on the optimal cut-off point (mortality risk of 0.7 %). Consequently, patients with a mild or moderate cholangitis according to TG13 are assigned to the high risk group, if the predicted mortality risk is 0.7 % or higher.

Occurrence of bacteraemia was also significantly associated with a higher mortality rate, stressing the importance of blood culture collection in patient with acute cholangitis, so that empirical antimicrobial treatment can be optimized in cases of bacteraemia. However, bacteraemia is not a good parameter to evaluate at the time point of hospital admission. In clinical practice it is not possible to diagnose bacteraemia immediately at the time point of hospital admission, because the current microbiological analysing methods used to detect pathogens in blood cultures still create a delay of at least one day. Major focus of the study was to create a risk prediction model that is easy to perform at the time point of hospital admission. Therefore, the predictor “bacteraemia” as well the predictor “PTC as interventional approach” were excluded from the model. Despite the exclusion of these two predictors, the AUC of the final model was excellent. Referring to the optimal cut-point, which is a predicted mortality risk of 0.7 %, the corresponding negative predictive value of the risk prediction model shows that it performs particularly well when it comes to the identification of patients that will survive. The positive predictive value is much lower which means that only a small fraction among the patients that are assigned a high mortality risk by the model will actually die. In the clinical setting and from a patient perspective such properties are beneficial as, firstly, the mortality rate in the low risk group is actually low. Consequently there is almost no danger of having high risk patients in this group. Secondly, the high risk group is defined wide enough to include all of the real high risk patients which is reflected by a high sensitivity. This comes at the expense of the inclusion of some patients with actual low risk, of course.

It is reasonable to triage patients with acute cholangitis according to their individual mortality risk. On the one hand, emergency interventions outside the routine operations are resource intensive and choice of antimicrobial agents, in particular reserve antibiotics should be selected with care in the face of increasing antimicrobial resistance development [26, 27]. On the other hand, early biliary decompression to restore biliary drainage and the administration of an effective antimicrobial treatment are essential for survival in patients with septic shock [20, 28]. Consequently, a feasible prediction model helping physicians to determine the time point of intervention and the choice of antimicrobial agents upon the patient’s risk for mortality would be desirable. Referring to the individual mortality risk calculated by our prediction model, we recommend urgent biliary drainage for patients with a high predicted mortality risk. In contrast, elective drainage may be appropriate for patients with a low predicted mortality risk presenting a mild grade of severity as we could show that the negative predictive value of our prediction model is very high. The proposed cut-point helps to allocate patients to a high and low risk group. However, this treatment algorithm requires prospective evaluation.

The strength of this study lies in the large sample size and the broad spectrum of clinical, laboratory, etiological factors which were evaluated. Major limitations of the study are its retrospective nature and single centre character, which limits the extrapolation of our data to other centres, although nested cross-validation was performed. A further limitation is that only the in-hospital mortality and not the 30 days mortality was assessed.

## Conclusion

Out of eleven investigated statistical models, a Random Forest model achieved excellent prediction performance (measured by AUC) and is provided online (http://www2.imse.med.tum.de:3838/) for individual risk prediction. Based on the predicted risk for mortality, we propose a simple treatment algorithm. However, this model requires prospective evaluation by multi-center studies.

### Ethics

The study was approved by the Ethics Committee, Klinikum rechts der Isar, Technische Universität München. Due to the retrospective study design, written consent was waived by the institutional review board.

## Abbreviations

MAC: Mortality in acute cholangitis
TG13: Tokyo Guideline 2013
ERC: Endoscopic retrograde cholangiography
PTC: Percutaneous transhepatic cholangiography
temp: Temperature
°C: Celsius
G/L: Giga/liter
CRP: C-reactive protein
mg/dL: Milligram/decilitre
AIC: Akaike’s Information Criterion
BIC: Bayesian Information Criterion
SVM: Support vector machines
CI: Confidence interval
OR: Odds ratio
AST: Aspartat-Aminotransferase
AUC: Area under the curve
%: Percent.
